# HTINet2: herb–target prediction via knowledge graph embedding and residual-like graph neural network

**DOI:** 10.1093/bib/bbae414

**Published:** 2024-08-23

**Authors:** Pengbo Duan, Kuo Yang, Xin Su, Shuyue Fan, Xin Dong, Fenghui Zhang, Xianan Li, Xiaoyan Xing, Qiang Zhu, Jian Yu, Xuezhong Zhou

**Affiliations:** Institute of Medical Intelligence, Department of Artificial Intelligence, Beijing Key Lab of Traffic Data Analysis and Mining, School of Computer Science & Technology, Beijing Jiaotong University, Beijing 100044, China; Institute of Medical Intelligence, Department of Artificial Intelligence, Beijing Key Lab of Traffic Data Analysis and Mining, School of Computer Science & Technology, Beijing Jiaotong University, Beijing 100044, China; Institute of Medical Intelligence, Department of Artificial Intelligence, Beijing Key Lab of Traffic Data Analysis and Mining, School of Computer Science & Technology, Beijing Jiaotong University, Beijing 100044, China; Institute of Medical Intelligence, Department of Artificial Intelligence, Beijing Key Lab of Traffic Data Analysis and Mining, School of Computer Science & Technology, Beijing Jiaotong University, Beijing 100044, China; Institute of Medical Intelligence, Department of Artificial Intelligence, Beijing Key Lab of Traffic Data Analysis and Mining, School of Computer Science & Technology, Beijing Jiaotong University, Beijing 100044, China; Institute of Medical Intelligence, Department of Artificial Intelligence, Beijing Key Lab of Traffic Data Analysis and Mining, School of Computer Science & Technology, Beijing Jiaotong University, Beijing 100044, China; Institute of Medical Intelligence, Department of Artificial Intelligence, Beijing Key Lab of Traffic Data Analysis and Mining, School of Computer Science & Technology, Beijing Jiaotong University, Beijing 100044, China; Institute of Medicinal Plant Development, Chinese Academy of Medical Sciences & Peking Union Medical College, Beijing 100193, China; Institute of Medical Intelligence, Department of Artificial Intelligence, Beijing Key Lab of Traffic Data Analysis and Mining, School of Computer Science & Technology, Beijing Jiaotong University, Beijing 100044, China; Institute of Medical Intelligence, Department of Artificial Intelligence, Beijing Key Lab of Traffic Data Analysis and Mining, School of Computer Science & Technology, Beijing Jiaotong University, Beijing 100044, China; Institute of Medical Intelligence, Department of Artificial Intelligence, Beijing Key Lab of Traffic Data Analysis and Mining, School of Computer Science & Technology, Beijing Jiaotong University, Beijing 100044, China

**Keywords:** drug–target prediction, network embedding, graph neural network, knowledge graph

## Abstract

Target identification is one of the crucial tasks in drug research and development, as it aids in uncovering the action mechanism of herbs/drugs and discovering new therapeutic targets. Although multiple algorithms of herb target prediction have been proposed, due to the incompleteness of clinical knowledge and the limitation of unsupervised models, accurate identification for herb targets still faces huge challenges of data and models. To address this, we proposed a deep learning-based target prediction framework termed HTINet2, which designed three key modules, namely, traditional Chinese medicine (TCM) and clinical knowledge graph embedding, residual graph representation learning, and supervised target prediction. In the first module, we constructed a large-scale knowledge graph that covers the TCM properties and clinical treatment knowledge of herbs, and designed a component of deep knowledge embedding to learn the deep knowledge embedding of herbs and targets. In the remaining two modules, we designed a residual-like graph convolution network to capture the deep interactions among herbs and targets, and a Bayesian personalized ranking loss to conduct supervised training and target prediction. Finally, we designed comprehensive experiments, of which comparison with baselines indicated the excellent performance of HTINet2 (HR@10 increased by 122.7% and NDCG@10 by 35.7%), ablation experiments illustrated the positive effect of our designed modules of HTINet2, and case study demonstrated the reliability of the predicted targets of Artemisia annua and Coptis chinensis based on the knowledge base, literature, and molecular docking.

## Introduction

The formulas of traditional Chinese medicine (TCM), characterized by its multi-component and multi-target effects, naturally excels in regulating complex diseases. It has been widely practiced in clinical diagnosis and treatment in China and is gradually gaining global application [1, 2]. The targets of drugs/herbs are the origin of a drug’s therapeutic action in the human body. Therefore, identifying herb–target interaction (HTI) is crucial as it aids in understanding the mechanisms of TCM, guiding rational clinical medication, and new drug development. Traditionally, the discovery of herb’s targets primarily relied on wet experiments or clinical trials. However, most herbs contain dozens to thousands of components, making the experimental technologies for discovering the targets of herb, which require simultaneous identification of effective components of herbs and their corresponding biological targets, extremely costly and time-consuming [3, 4]. In recent years, with the advancement of informatics technology and the accumulation of a vast amount of biomedical data, computational techniques such as machine learning and deep learning are increasingly used to predict the targets of herbs, becoming a mainstream approach [5]. Compared with traditional methods, computational prediction significantly reduces the cost of target discovery, enhances prediction accuracy, and accelerates the pace of new drug development [6].

In recent years, researchers have designed various computational methods for predicting the targets of herbs, primarily employing techniques such as network propagation, network embedding, and random walks among other machine learning algorithms. Yang *et al*. [7] developed a network propagation-based model for herb–target prediction (HTP), motivated by the idea that herbal targets with similar efficacy are also similar. This model used the efficacy-based herb similarity to initialize the values of node in protein–protein interaction (PPI) network, and employs random walks on the network to score candidate proteins. Additionally, Yang *et al*. [8] proposed a model named heNetRW, which leverages random walks on a heterogeneous herb–target network, demonstrating commendable predictive performance. Wang *et al*. [9] introduced an HTP method named HTINet based on heterogeneous network embedding. They construct a heterogeneous network of symptoms–herbs–targets, and use network embedding to learn low-dimensional vectors of herbs and proteins, ultimately achieving precise target prediction.

Furthermore, with the advancement of deep learning, various drug prediction methods based on deep neural networks have been proposed. Zhang *et al*. [10] introduced a multi-view deep learning model, DrugAI, which combines graph neural networks, network embedding, and multi-view learning to predict activation–inhibition relationships between drug and target. Yang *et al*. [11] proposed a drug repositioning algorithm named DRONet based on network embedding and ranking learning, aimed at discovering new indications for drugs. Lin *et al*. [12] developed an end-to-end algorithm based on deep neural networks, named GraphCPI, which employs graph neural networks to predict the targets of compounds. Concurrently, several pharmacological databases have been developed. Mohanraj *et al*. [13, 14] constructed the IMPPAT digital database, which focuses on the phytochemicals of Indian medicinal plants and integrates information from traditional books and published research articles. Wu *et al*. [15] developed SymMap, an integrative database of TCM enhanced by symptom mapping. SymMap aligns TCM with modern medicine at both the phenotypic and molecular levels in shared aspects. These pharmacological databases support and facilitate the discovery of drug–target relationships.

Although various prediction algorithms of drug/herb’s targets have been proposed, HTI prediction task still faces several challenges. For example, the existing HTI models do not make full use of TCM knowledge. On the one hand, HTI relationships are closely associated with the TCM properties of herbs, such as their property, flavor, and meridian, as well as their efficacy. On the other hand, in clinical practice, the therapeutic action of herbs is manifested through the diseases and symptoms they treat. However, to date, only HTINet [9] has considered the information of diseases and symptoms in target prediction, but it does not take into account the TCM properties of herbs. Therefore, integrating both the TCM properties and clinical treatment of herbs into the HTI modeling to achieve more accurate predictions remains a challenge.

Additionally, the existing models predict HTI relationships primarily using unsupervised learning techniques, such as random walks and network embedding [8, 9]. These methods are unable to use known HTI as the supervisory information of model and fail to capture the full spectrum of known interactions, which hinders further improvement in prediction accuracy. Therefore, designing more efficient supervised deep learning models to enhance the accuracy of HTI poses another challenge.

In this study, we proposed a deep learning-based HTP framework termed HTINet2, which includes three key modules, i.e. TCM knowledge graph embedding (KGE), residual graph representation learning, and supervised target prediction. In the module of KGE, we constructed a knowledge graph (termed TMKG) that fuses the TCM property and clinical treatment knowledge of herbs, and learned the deep knowledge embedding (KE) of herbs and targets. In the module of graph representation learning and target prediction, we designed a residual graph convolution neural network to capture the deep interactions among herbs and targets, and a Bayesian personalized ranking (BPR) to achieve supervised model training. Finally, we designed comprehensive experiments including performance comparison, ablation experiments, hyper-parameter analysis, and case study, which indicated the excellent performance and the reliability of predicted results of HTINet2.

## Materials and methods

### Dataset

#### Herb–target interactions

The data of HTIs are derived from SymMap [15], which is a known database centered on symptoms, herbs, and molecular. First, we downloaded all the HTI, totaling 267 809 relationships. To ensure the reliability of relationships, we filtered out a relation subset based on an inferred evidence score exceeding the average value of 0.4616 and a *P*-value ¡0.05. Finally, we obtained a benchmark dataset of 38 002 HTI relationships, including 563 distinct herbs and 2106 distinct targets.

#### Knowledge graph of TCM and western medicine (TMKG)

To make full use of medical knowledge in modeling HTI prediction, we constructed a knowledge graph of TCM and western medicine through the extraction and integration from multiplex biomedical knowledge bases and TCM books. These databases include SymMap [15], soFDA [16] centered on the ontology of TCM syndromes, STRING [17] v11 including large-scale PPI, KEGG [18] of genomic encyclopedia, and GO of gene ontology [19]. The TCM books consist of ”Pharmacopoeia of the People’s Republic of China 2020”, ”100 Classic Famous Formulas”, ”Traditional Chinese Medicine Diagnostics”, ”Treatise on Febrile Diseases”, ”Formulas of Traditional Chinese Medicine”, and ”Internal and External Women and Children’s Diseases”. To ensure the quality of our constructed knowledge graph, we used programs or scripts for data cleaning, and we invited medical experts to review and correct the data, as detailed in Section 1.1 of Supplemental Materials file 1.

Finally, the TMKG we constructed comprises 15 types of 74 529 entities, including herbs, efficacy, meridian, categories, diseases, components, properties, symptoms, syndromes, prescriptions, Gene Ontology (GO), pathways, and various levels of syndromes. It includes 77 008 head entities and 130 402 tail entities, spanning 31 types of relations such as herb-efficacy, herb-symptom, herb-prescription, herb-ingredient, herb-property, and herb-meridian, totaling 1920 415 triples (Section 1.1 of Supplemental Materials file 1).

### HTINet2 framework to predict herb’s targets

#### Overall architecture of HTINet2

**Figure 1:**
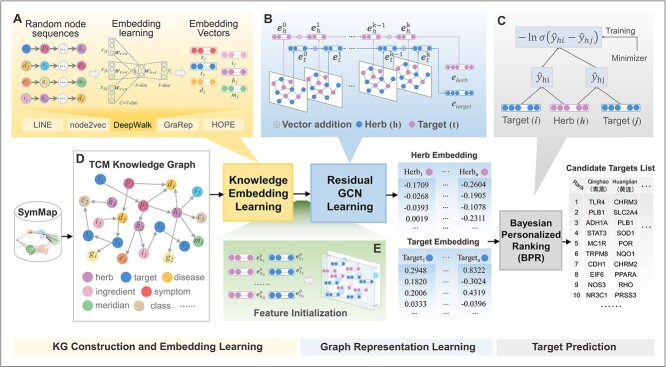
Overall architecture of HTINet2; HTINet2 consists of three key modules, i.e. KG construction and embedding learning (A and D), graph representation learning (B and E), and target prediction (C).

We proposed HTINet2 (Fig. 1), a HTI prediction framework, which comprises three key modules, namely KG construction and embedding learning, graph representation learning, and target prediction. In the first module, we construct a knowledge graph TMKG centered on molecular, TCM theories and clinical treatments of herbs. Then we applied KE learning algorithms to learn the complex relationships among entities in TMKG, resulting in their respective embedding vectors. In the second module, we constructed a heterogeneous network specifically tailored for target prediction, introducing a learning method of residual graph convolutional network (GCN). This supervised method builds upon KE features to further learn intensified representation of herbs and proteins. In the final module of target prediction, we implement a BPR loss function to optimize the parameters of residual GCN, facilitating the prediction of potential targets.

#### KE learning of TMKG

To obtain implicit knowledge of the TMKG, we utilized KE algorithms to learn the embedding representations of all entities within the KG. KE algorithms are adept at learning the latent relationships between various entities in the KG (i.e. implicit local features) as well as the overall network structure of the KG (i.e. implicit global features). Subsequently, the learned implicit features are presented as the embedding vectors of nodes, which can be used for downstream tasks such as classification or link prediction, particularly predicting HTI in this study.

Among various KE algorithms, DeepWalk is particularly noteworthy. DeepWalk uses random walks to explore the KG, thereby learning a sequence of nodes which approximates the structural context of each node. Given a graph $G=(V,E)$, where $V$ represents the set of vertices, and $E\subseteq (V\times V)$ represents the set of edges, the v denotes a node in the set of vertices $V$. The loss function of DeepWalk is mathematically formulated as 


(1) \begin{align*} & \mathcal L = -\log\Pr(\{v_{i-w},...,v_{i+w}\} \setminus v_{i} \mid \Phi(v_{i}));\end{align*}

(2) \begin{align*} & \Pr(\{v_{i-w},...,v_{i+w}\} \setminus v_{i} \mid \Phi(v_{i})) = \prod_{j=i-w \ j\ne i}^{i+w}\Pr(v_{j} \mid \Phi(v_{i})),\end{align*}

where $\mathcal L$ is the loss function, $v_{i}$ is the current node in the graph, $\{v_{i-w},...,v_{i+w}\}$ represents the neighborhood of the current node, and $w$ is the size of the window. $\Phi (v_{i})$ is the function mapping nodes to their vector embeddings (as detailed in the Section 1.2 of Supplemental Materials file 1).

Additionally, owing to the impact of different KE algorithms and embedding dimensions on these downstream tasks, we compared five KE algorithms, namely LINE [20], GraRep [21], node2vec [22], DeepWalk [23], and HOPE [24], along with different dimensions (i.e. 32, 64, 96, 128, 256, and 512). Ultimately, the most effective KGE algorithms and embedding dimensions for the task of HTI prediction are selected.

#### Graph representation learning for HTI network

Graph representation learning, particularly GCNs, has emerged as one of the key methodologies for learning and representing complex relationships within graph-structured data. To fully leverage the intricate interactions between herbs and targets, thereby forming more precise representation vectors of herbs and targets, we have developed a residual-like graph convolutional neural network [25]. This network takes the HTI network as its initial input, with the embedding vectors derived from KE learning module serving as the initial node features. Interaction information of herbs and targets could be propagated through residual-like GCN operations, ultimately learning deeper representation features of them.

Formally, based on the known drug–target relationships in the training set, we constructed a heterogeneous drug–target graph $G\!=\!\left \langle V,A \right \rangle $, where $V$ is the set of nodes (including herbs and targets), and the adjacency matrix $A$ encodes the interaction relationships between herbs and targets. In this matrix, if a herb $i$ has an interaction with target $j$, then $a_{\mathrm{ij}}\!=\!1$, otherwise, it is 0. Subsequently, we computed the normalized matrix $S={\widetilde{D}}^{\mathrm{-0.5}}\widetilde{A}{\widetilde{D}}^{\mathrm{-0.5}}$, to weight the influence of each node’s degree, where $\widetilde{A}=A+I$ represents the adjacency matrix with self-loops ($I$ being the identity matrix), and $\widetilde{D}$ is the degree matrix of $\widetilde{A}$.

In the multi-layer GCN we constructed, if the node feature matrix at the $k$-th layer is denoted as $E^{k}$, then after the propagation and learning of node features, the features at the $(k+1)$-th layer can be represented as 


(3) \begin{align*}& E^{k+1}={\widetilde{D}}^{-0.5}\widetilde{A}{\widetilde{D}}^{-0.5}E^{k}W^{k},\end{align*}

where $W^{k}$ represents the weight matrix; it adjusts the relative importance of different input variables and is continuously optimized during the training process of neural networks. To maintain the integrity of neighborhood information, and reduce computational costs, we replaced the nonlinear activation functions in each layer with linear activation functions for feature propagation. In the HTI network, the node degrees of an herb $h$ and a target $t$ are denoted as $d_{h}$ and $d_{t}$, respectively. Their respective neighbor node sets are represented as $R_{h}$ and $R_{t}$. Their vector representations in the $k$-th layer of the graph neural network are $e_{h}^{k}$ and $e_{t}^{k}$. Consequently, after one layer of GCN feature propagation, their representations at the $(k+1)$-th layer can be obtained: 


(4) \begin{align*} e_{h}^{k+1} &= \left[ \frac{1}{d_{h}} e_{h}^{k} + \sum_{t \in R_{h}} \frac{1}{d_{t} \times d_{h}} e_{t}^{k} \right] W_{k} \end{align*}

(5) \begin{align*} e_{t}^{k+1}& = \left[ \frac{1}{d_{t}} e_{t}^{k} + \sum_{t \in R_{t}} \frac{1}{d_{t} \times d_{h}} e_{h}^{k} \right] W_{k} \end{align*}

Overly deep network architectures may lead to issues of gradient vanishing or explosion in GCN. Inspired by the residual operation of ResNet, we obtained the final embedding vectors of herbs/targets, $e_{h}$ and $e_{t}$, by fusing the vectors of after k layers graph convolutional operations, $e_{h}^{1},e_{h}^{2}\ldots e_{h}^{k-1},e_{h}^{k}$ and $e_{t}^{1},e_{t}^{2}\ldots e_{t}^{k-1},e_{t}^{k}$, along with the initial node representations $e_{h}^{0}$ and $e_{t}^{0}$. 


(6) \begin{align*} e_{h}&=e_{h}^{0}+e_{h}^{1}+\cdots+e_{h}^{k-1}+e_{h}^{k} \end{align*}

(7) \begin{align*} e_{t}&=e_{t}^{0}+e_{t}^{1}+\cdots+e_{t}^{k-1}+e_{t}^{k} \end{align*}

The residual-like feature aggregation operation captures more complex features of the GCN by considering the feature outputs at each layer, and makes the information transfer between network layers more efficient, finally obtaining more precise representations of herbs and targets.

#### Herb–target prediction

Building upon graph representation learning module, we designed an optimization loss of BPR for predicting HTI relationships. As a commonly used method in the field of recommendations, BPR loss facilitates learning the interactions between herbs and targets by maximizing the margin between positive and negative samples. This margin is assessed by comparing the scores of herb–target pairs, where the score signifies the level of similarity or matching of a herb–target pair.

Mathematically, for a triple $(h,t_{i},t_{j} )$, h represents an herb, $t_{i}$ a target associated with the herb (i.e. positive sample, composed of targets associated with the herb in the training set), and $t_{j}$ a target not associated with the herb (i.e. negative sample, composed of several negative samplings of positive samples). We utilized vector distance, simplified to dot product in this study, to calculate the scores for both the positive and negative sample pairs based on the embedding vectors of herbs and targets. We then constructed a pairwise ranking-based objective loss function, which seeks to maximize the score of positive samples while minimizing that of negative samples, thereby updating and optimizing the parameters of the neural network, as follows: 


(8) \begin{align*}& \min_{\Theta}\mathcal{L} = (\mathbf{R}, \hat{\mathbf{R}}) = \sum_{h=0}^{M} \sum_{(t_{i},t_{j}) \in D_{h}} -\ln(s(\hat{r}_{ht_{i}} - \hat{r}_{ht_{j}})) + \lambda ||\Theta||^{2}\end{align*}

Here, $s(x)$ signifies the Sigmoid function, $D_{h}$ encompasses all the HTI triples in the training set, $\Theta $ represents all the parameters for neural network, $||\Theta ||^{2}$ is the regularization term for the parameters, $\lambda $ denotes the weight of the regularization term, and M is the number of herbs. Ultimately, based on the trained neural network, score for each drug target pair can be obtained, allowing for the ranking prediction of targets based on these scores.

### Experimental settings

In our experiments, we divided the 38 002 HTI relationships into a training set, validation set, and test set in an 8:1:1 ratio. We compared HTINet2 with two categories of baseline methods. The first is the methods of traditional link prediction based on common neighbors, including CN, Salton [26], Jaccard [27], HPI [28], LHN-1 [29], AA [30], and RA [31]. The other comprises previously proposed models for HTI prediction, such as heNetRW [8] and Prince [32]. Detailed introduction for these baselines is described in the Section 1.3 of Supplemental Materials file 1. Furthermore, we selected hit ratio (HR) and normalized discounted cumulative gain (NDCG) as the evaluation metrics [33]. For a given herb $h$, $P_{h@K}$ represents the top-K predicted candidate genes, and $G_{h}$ represents the known genes of $h$ in the test set. HR@K is given by 


(9) \begin{align*}& HR@K=\frac{\left|P_{h@K}\bigcap G_{h}\right|}{\left|G_{h}\right|}\end{align*}

The Normalize Discounted Cumulative Gain (NDCG@K) can be calculated as follows: 


(10) \begin{align*}& NDCG@K=\frac{DCG@K}{IDCG@K}=\frac{\sum_{i=1}^{k}\frac{2^{r_{i}}-1}{\log_{2}{(i+1)}}}{\sum_{i=1}^{\left |REL\right | }\frac{2^{r_{i}}-1}{\log_{2}{(i+1)}}},\end{align*}

where DCG represents Discounted Cumulative Gain, IDCG represents Ideal Discounted Cumulative Gain, $r_{i}$ represents the relevance score of the gene at position $i$ in the ranked list, and $\left | REL \right | $ represents the results sorted by relevance.

## Results

### TMKG and KE

**Figure 2:**
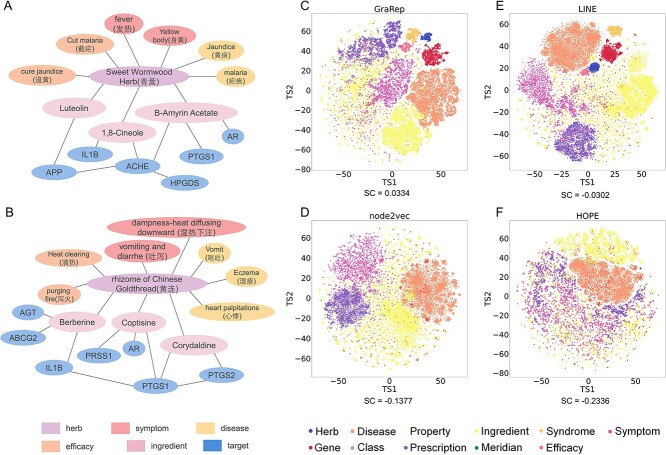
(**A** and **B**) Schematic representation centered on Artemisia annua and Coptis in knowledge graph TMKG; (**C**–**F** visualization of embedding vectors of entities in TMKG by different KE methods.

We constructed a large-scale knowledge graph of Traditional Chinese and Modern Medicine TMKG by the information extraction and curation from multiple public databases. The TMKG comprises 15 types of entities totaling 74 529 and 31 types of triples totaling 1920 415. For subsequent HTP, TMKG included two types of vital information related to herbs. The first is the TCM properties of herbs, including herbal property and class, taste, meridian, and efficacy, etc. The second is the clinical treatment information of the herbs, including diseases and symptoms. As shown in Fig. 2A and B, we took Artemisia annua and Coptis as examples, and illustrated their connections to syndromes, diseases, efficacy, ingredients, and proteins.

**Table 1:** Performance comparison of HTP methods.

	**Top@1**		**Top@3**		**Top@5**		**Top@10**	
**Models**	**HR**	**NDCG**	**HR**	**NDCG**	**HR**	**NDCG**	**HR**	**NDCG**
CN	0.0274	0.0812	0.0799	0.1705	0.1206	0.2131	0.2001	0.276
Salton	0.0097	0.0294	0.0284	0.0614	0.0438	0.0792	0.0805	0.1117
Jaccard	0.0113	0.0305	0.0335	0.0667	0.053	0.085	0.0983	0.1255
HPI	0.0003	0.0006	0.003	0.0073	0.0067	0.0162	0.0201	0.04
LHN-1	0.0003	0.0005	0.0012	0.0015	0.0022	0.0031	0.0062	0.0079
AA	0.0278	0.0831	0.0787	0.1669	0.1209	0.2148	0.1996	0.2756
RA	0.027	0.0795	0.0763	0.1569	0.117	0.2118	0.1929	0.2667
heNetRW	0.0080	0.0186	0.0254	0.0436	0.0455	0.0597	0.0915	0.0942
PRINCE	0.0032	0.0065	0.0134	0.0174	0.0269	0.0282	0.0495	0.0422
**HTINet2(ours)**	**0.3536**	**0.3536**	**0.3448**	**0.3398**	**0.3706**	**0.35**	**0.4458**	**0.3746**

To capture the complex and deep relationships among biological entities (especially herb and target) in TMKG, we utilized multiplex methods of KE to learn the embedding representations of entities, which imply extensive and accurate medical knowledge, e.g. the TCM properties and clinical treatment information of herbs. These embedding vectors encapsulate information pertaining to the current node and its neighboring nodes within the context of a complex knowledge graph. Then we used t-SNE [34] to reduce the dimension of the embedding vectors of these entities learned by different KE models, and visualize them on a two-dimensional plane. We calculated the Silhouette score (SC) for various entities, as shown in Fig. 2C–F and the Section 2.1 of Supplemental Materials file 1. These entities, including herbs, proteins, symptoms, and diseases, etc. are grouped into different clusters.

Second, the results also showed that there are obviously differences on the embedding visualization of KE methods with different model characteristics. For example, in the visualization results of GraRep, the cluster distance between herbs and diseases, genes, and efficacy are relatively close, while the distance between herbs and ingredients was relatively far away. For the result of LINE, the herb cluster was in the center; in addition to the distance from the herbal prescription, it has a relatively close distance from other entities’ cluster. Finally, the KE model can capture the complex relationship between entities and get a good embedding representation, which provides a basis for fine-tuning the downstream task of modeling HTI prediction.

### Overall performance comparison with baselines

In the experiments, we compared the performance of HTINet2 with multiple baseline methods, which include traditional link prediction algorithms and the known HTI prediction methods, i.e. heNetRW and PRINCE.

**Figure 3:**
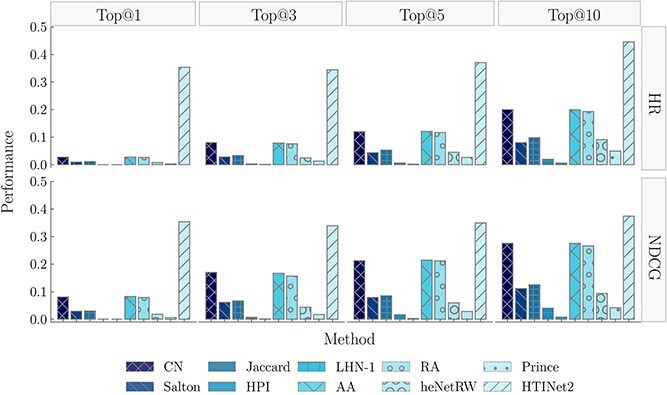
Performance comparison of HTP methods.

The experimental results (Table 1 and Fig. 3) showed that in the baseline of local similarity, CN and AA, which are both network topology-based methods, achieved the best performance (HR@5=0.1209 and NDCG@10=0.276), followed by RA as well, and the HPI and LHN-1 performed worst. In the two known baselines, heNetRW obtained the highest performance (HR@10=0.0915 and NDCG@10=0.0942). The baseline methods based on local similarity most often outperformed the two known baselines. For example, the HR@10 and NDCG@10 of CN are 118.7% and 193.0% higher than those of heNetRW, respectively, indicating that local similarity methods can effectively capture the structural information of HTI network, demonstrating superior performance in HTI prediction.

By comparing the performance of HTINet2 with all baseline methods, the results showed that HTINet2 achieves the best performance (HR@10=0.4458 and NDCG@10=0.3746), which are significantly higher than all baseline methods. For example, the HR@10 and NDCG@10 metrics of HTINet2 are 122.8% and 35.7% higher than those of the best baseline (i.e. CN). The excellent performance of HTINet2 is benefit from the pre-training of KE learning and the fine-tuning of residual-like GCN.

**Table 2:** Performance comparison of key modules of HTINet2.

	**Top@1**		**Top@3**		**Top@5**		**Top@10**	
**Models**	**HR**	**NDCG**	**HR**	**NDCG**	**HR**	**NDCG**	**HR**	**NDCG**
HTINet2	0.3536	0.3536	**0.3448**	**0.3398**	**0.3706**	**0.35**	**0.4458**	**0.3746**
HTINet2 w/o PT	**0.3556**	**0.3556**	0.3389	0.3389	0.3663	0.3447	0.435	0.3727
	($\uparrow $0.57%)	($\uparrow $0.57%)	($\downarrow $1.71%)	($\downarrow $0.26%)	($\downarrow $1.16%)	($\downarrow $1.51%)	($\downarrow $2.42%)	($\downarrow $0.51%)
HTINet2 w/o RES&PT	0.3285	0.3285	0.3169	0.3172	0.3331	0.32	0.3972	0.3433
	($\downarrow $7.62%)	($\downarrow $7.62%)	($\downarrow $6.49%)	($\downarrow $6.40%)	($\downarrow $9.06%)	($\downarrow $7.17%)	($\downarrow $8.69%)	($\downarrow $7.89%)

**Figure 4:**
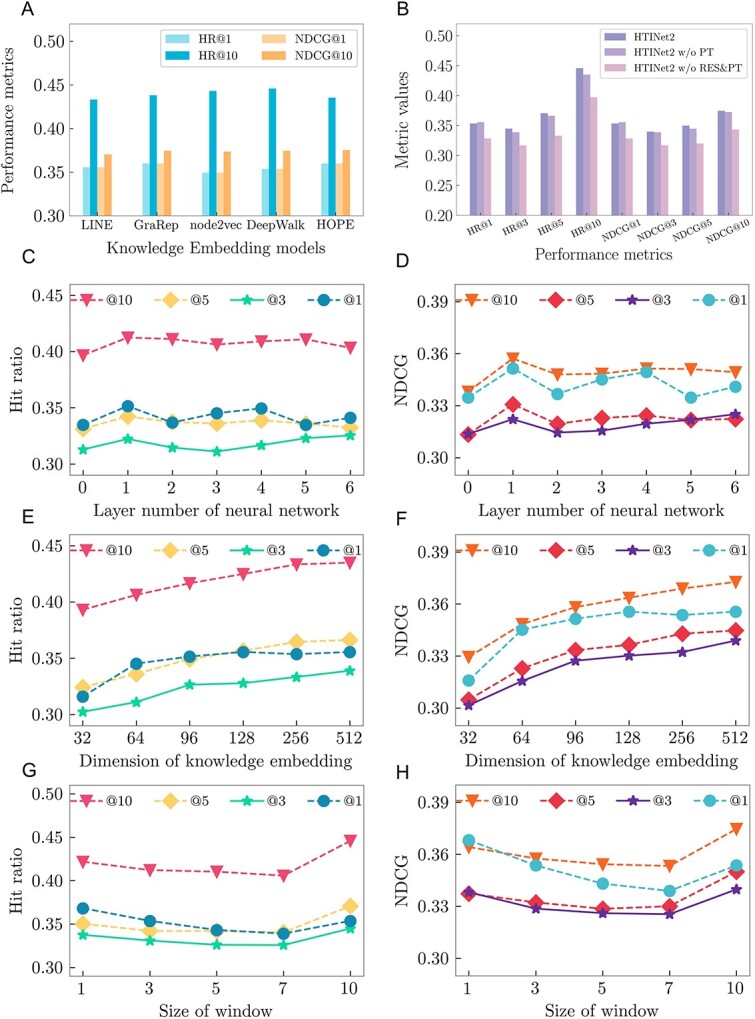
Performance comparison and parameter sensitivity analysis of HTINet2; (**A**) performance comparison of different KE methods; (**B**) performance comparison of different components of HTINet2; (**C** and **D**) HTINet2’s performance with different numbers of GCN layers; (**E**–**F**) HTINet2’s performance with different dimensions of KE; (**G**–**H**) HTINet2’s performance with different window size.

To obtain deep knowledge representations for herbs and targets, we utilize different algorithms of pre-training KE to learn the pre-training features of herbs and targets from the TMKG. As shown in Fig. 4 and the Section 2.2 of Supplemental Materials file 1, the results indicate that the pre-training of HTINet2 has a good robustness that all pre-training methods improve prediction performance compared with non-pre-training. Specifically, DeepWalk and HOPE exhibit the best performance in HTI prediction. This may be attributed to the graph structure of TMKG; both DeepWalk and node2vec employ random walk to capture the structural information. However, the uniform random walk used in DeepWalk might more accurately reflect the true structure of the graph in the context of HTI prediction tasks. In addition, HOPE is designed to capture high-order proximities in a graph. Capturing deep interactions in the HTI network may be more crucial than considering direct neighbors alone. We have presented the target predictions for all drugs in the dataset, predicted by the HTINet2 model, in the Supplemental Materials file 2.

### Performance influence of key components in HTINet2

To evaluate the contribution of different components in HTINet2, we performed ablation experiments of HTINet2, including KE pre-training (termed PT) and residual GCN (termed RES). The results (Table 2 and Fig. 4B) showed that, except in rare metrics (i.e. HR@1 and NDCG@1), the removal of any components from HTINet2 leads to a performance decline in the most terms of metrics. Specifically, the performance decreases the most (HR@10 by 8.69% and NDCG@10 by 7.89%) when removing the residual and KG pre-training components simultaneously. The performance decreases the least (HR@10 reduced by 2.42% and NDCG@10 by 0.51%) when removing the pre-training component. This indicates that each component of HTINet2 contributes to its performance, with the contribution of the residual in GCN contributing more than the KG pre-training.

Above results indicate that pre-training of TMKG enhances the expressive capabilities of subsequent components. Moreover, the interactions between herbs and genes within the KG provide additional context for the model. By utilizing the effective information transformed through residual-like GCN, the model is better equipped to capture the latent characteristics of the nodes, improving the prediction performance.

### Interpretability of HTI prediction model

**Figure 5:**
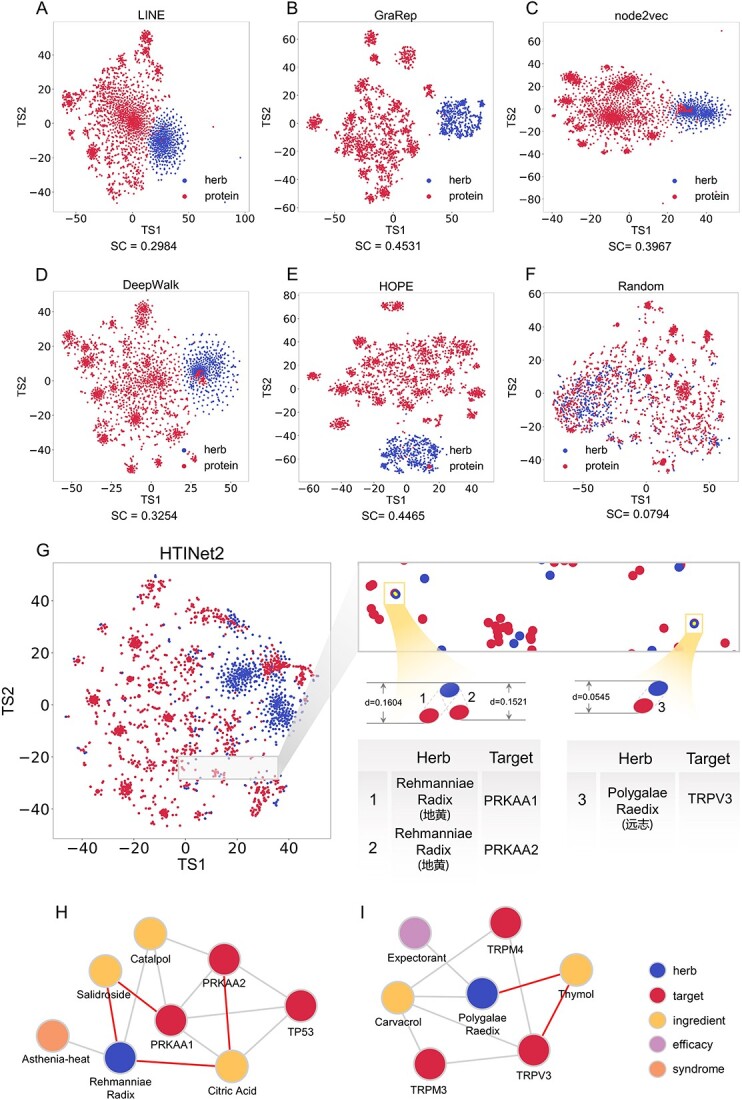
(**A**–**E**) Visualization of the pretrained features of herbs and targets in TMKGs by different KE methods; (**F**) visualization of embedding features of herbs and targets initialized from normal distribution; (**G**) visualization of embedding features of herbs and targets by HTINet2 and several examples of HTI; (**H**) relationships between Rehmanniae Radix, PRKAA1, and PRKAA2 in TMKG; (**I**) relationships between Polygalae Radix and TRPV3 in TMKG.

To investigate the interpretability of HTI prediction models, we used t-SNE [34] to reduce the dimension of the embedding vectors of herbs and targets learned by different prediction models, and visualize them on a two-dimensional plane. First, the visualization result (Fig. 5A–F) indicated that five KE methods (i.e. LINE, GraRep, node2vec, DeepWalk, and HOPE) effectively divided herbs and targets into two distinct clusters, showing a clear advantage over random embedding vectors. The results of SC for these clusters indicate there are high SC of GraRep, DeepWalk, and HOPE, of which two (HOPE and DeepWalk) also achieve high prediction performance. This indicates there is a positive correlation between performance of HTINet2 and clustering of KE models.

To intuitively display and further explain the predicted results of HTINet, we also showed the visualization result (Fig. 5G) after the KE and residual-like GCN of HTINet2. Then we selected some HTI relationships in which herbs and targets are very close in a two-dimensional plane. For example, the Rehmanniae Radix is close to two known targets, PRKAA1 and PRKAA2, with distances of 0.1604 and 0.1521, respectively. The two targets PRKAA1 and PRKAA2 that almost coincide in position are also proteins from the same family. There is a closer distance (0.0545) between the Polygalae Radix and its target TRPV3. We revisited the TMKG, analyzing the selected HTI relationships and the neighbors of these entities. Figure 5H-I demonstrates these relationships within the TMKG we constructed, where entities with closer vector distances show strong relevance. We have highlighted some possible paths with red lines. For example, the second-order neighbors of Rehmanniae Radix are PRKAA1 and PRKAA2, and those of Polygalae Radix are TRPV3. On the whole, most herb targets with existing or potential relationships are closely spaced in the embedding visualized results of our HTINet2.

### Hyper-parameter sensibility of HTINet2

To evaluate the robustness of HTINet2, we conducted sufficient experiments on hyper-parameter sensitivity analysis. In experiments, the main hyper-parameters of HTINet2 include the number of GCN layers in the Graph Representation Learning module, the embedding dimension of entities (e.g. herbs and targets), and the window size of the DeepWalk in KE learning stage.

When conducting experiments on the number of GCN layers in the component of Graph Representation Learning, we fixed the dimension of embedding vector at 64. The results (Fig. 5C-D, Table 3 of Supplemental Materials file 1) show a noticeable impact of layer quantity on the performance of HTINet2. Specifically, as the number of layers increased, there was a significant improvement in the metrics of HR and NDCG, followed by a reduction and then a stable trend thereafter.

We fixed the number of GCN layers and conducted experiments by adjusting the embedding vector dimensions. The results (Fig. 4E-F, Table 4 of Supplemental Materials file 1) indicate that the performance of our model gradually improved across all metrics with the increase in embedding vector dimensions. This improvement can be attributed to the fact that higher dimensional vectors encapsulate more information, enabling a more comprehensive representation of herbs and targets, improving the prediction performance of HTINet2. Finally, we adjusted the window size of the DeepWalk method during the KE learning stage, and the results (Fig. 4G-H, Table 5 of Supplemental Materials file 1) show that the model exhibits slight sensitivity to the window size. As the window size increases, the overall performance of the model exhibits a declining trend, and there is a slight rebound in performance when the window size is set to 10.

### Case study

To demonstrate the reliability of HTINet2’s prediction results, we selected the two herbs, namely Artemisia annua and Coptis chinensis, and showed the results of top 15 targets predicted by HTINet2, excluding the targets that were existing in the test set. To verify the potential relationships between herbs and their predicted targets, we conducted three types of validation, including database, literature and molecular docking. Database validation is based on the two authoritative Chinese herb databases, namely ETCM [35] and HERB [36]. Literature validation is based on newly published medical literatures from PubMed to identified possible associations. In docking validation, we selected the key compounds of Artemisia annua and Coptis chinensis, namely artemisinin and berberine, and conducted docking-based virtual screening (using molecular docking software Autodock 1.5.7) to investigate the potential relationships between these predicted targets and the two compounds. As detailed in Table.3, the targets highlighted in bold are those that have been verified based on three types of evidences.

**Table 3:** Prediction analysis of Artemisia annua and Coptis chinensis.

	**Artemisia annua( 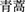 **)		**Coptis chinensis( 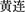 **)	
**Rank**	**Predicted target**	**Target validation**	**Predicted target**	**Target validation**
1	**TLR4**	ETCM	**CHRM3**	Docking (BE=-5.99)
2	PLB1	–	**SLC2A4**	Tang *et al.*
3	**ADH1A**	Docking (BE=-6.56)	PLB1	–
4	**STAT3**	Gao *et al.*	SOD1	–
		Ilamathi *et al.*		
5	**MC1R**	Docking (BE=-6.58)	**POR**	Docking (BE=-6.61)
6	**TRPM8**	ETCM	**NQO1**	Shou *et al.*
7	**CDH1**	Xu *et al.*	**CHRM2**	HERB and ETCM
8	**EIF6**	HERB	**PPARA**	Docking (BE=-7.61)
9	NOS3	–	**RHO**	Tang *et al.*
10	**NR3C1**	Docking (BE=-8.15)	**PRSS3**	Docking (BE=-6.9)
11	**EGFR**	Liu *et al.*	AHSA1	–
12	**CD86**	Docking (BE= -6.34)	**IL18**	Huang *et al.*
13	**AHR**	Wang *et al.*	GCLC	–
14	**MYC**	Hu *et al.*	MYC	–
15	**IL2**	Yang *et al.*	PSMD3	–

Note: Target validation including three types, namely database, literature, and molecular docking. Database validation is based on the two authoritative Chinese herb databases, namely ETCM and HERB. Literature validation is based on newly published medical literatures from PubMed. Docking validation is based on docking-based virtual screening, and BE denotes the binding energy.

**Figure 6:**
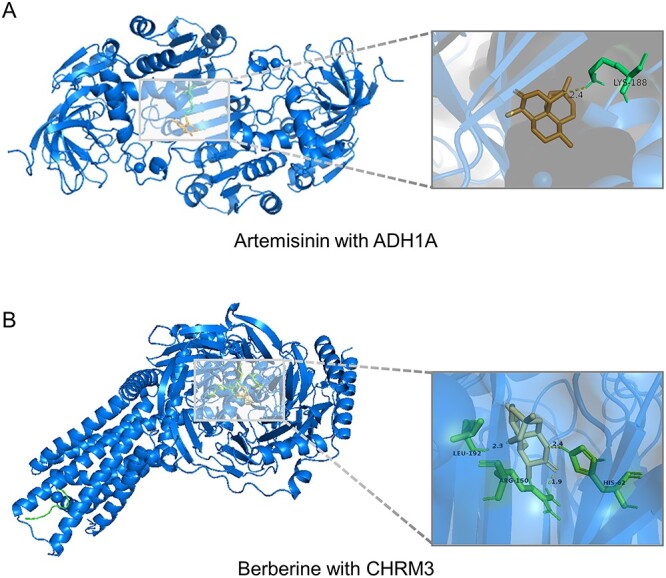
3D diagrams of molecular docking results; (**A**) artemisinin docked onto the amino acid residue of ADH1A, namely LYS-188; (**B**) berberine docked onto the three amino acid residues of the protein, namely HIS-62, ARG-150 and LEU-192.

- **Target validation of Artemisia annua.** In the top 15 candidate targets of Artemisia annua, evidence in databases, literature or docking supports 13 of these targets. The ETCM database developed by Zhang *et al*. [35] recorded TLR4 (ranked first) and TRPM8 (ranked sixth) as the targets of Artemisia annua. The HERB database studied by Fang *et al*. [36] recorded EIF6 (ranked eighth) as a target of this herb. Gao *et al*. [37] have explored that Dihydroartemisinin inhibits endothelial cell tube formation by suppression of the STAT3(ranked fourth) signaling pathway. Additionally, Ilamathi *et al*. [38] have demonstrated Artesunate, an anti-cancer agent, targets STAT3 and effectively suppresses hepatocellular carcinoma. Liu *et al*. [39] found that EGFR (ranked 11th) is related to the treatment of cervical cancer; oral administration of Dihydroartemisinin for 28 days reduced the expression of p53, EGFR, and Ki-67 antigens. Furthermore, several studies [40–43] have reported potential targets such as CDH1, AHR, MYC, IL-2, which may be associated with Artemisia annua or its derivatives. In addition, docking results indicated that there is strong binding energy with artemisinin for the four potential targets, namely ADH1A (ranked third, BE=-6.56), MC1R (ranked fifth, BE=-6.58), NR3C1 (ranked 10th, BE=-8.15), and CD86 (ranked 12th, BE=-6.34). For example, artemisinin docked onto the amino acid residue of ADH1A, namely LYS-188 (Fig. 6A). Other results of docking analysis are shown in the Section 2.4 of Supplemental Materials file 1.
- **Target validation of Coptis chinensis.** In the results of the top 15 candidate targets of Coptis chinensis, the evidence support nine of these targets. The study of Tang *et al*. [44] demonstrated that berberine, a major component of Coptis chinensis, may improve insulin resistance by affecting the expression of the SLC2A4 gene (ranked second) and increasing GLUT4 levels. Shou *et al*. [45] found that berberine activates PPAR$\delta $, initiating transcriptional regulatory functions and promoting the expression of NQO1 (ranked sixth). The CHRM2 gene, a target for the Coptis chinensis, is retrievable in both the HERB and ETCM databases. Additionally, several studies [46, 47] have reported potential targets, such as RHO and IL18, which may be related to Coptis chinensis or its derivatives. In addition, the results of docking showed there is strong binding energy with berberine for the four potential targets, including CHRM3 (ranked first, BE=-5.99), POR (ranked fifth, BE=-6.61), PPARA (ranked eighth, BE=-7.61), and PRSS3 (ranked tenth, BE=-6.9). Taking the potential target CHRM3 as an example, berberine docked onto the three amino acid residues of the protein, namely HIS-62, ARG-150, and LEU-192 (Fig. 6B). Other results of docking analysis are shown in the Section 2.4 of Supplemental Materials file 1.

The above case results indicated that our HTINet2 can obtain the reliable prediction results, capable of not only identifying existing drug targets but also suggesting new candidate targets for which evidence has not yet been found.

## Discussion

Benefiting from the efficiency of computation methods, computational prediction of drug targets has emerged as a hot topic. In this study, we proposed a deep learning framework to predict herbal targets, which includes TCM and clinical KGE, residual graph representation learning, and supervised target prediction. The comprehensive experiments and case study showed the excellent performance and the reliability of predicted results of HTINet2.

The high performance of HTINet2 benefits primarily from two points. On the one hand, the knowledge graph TMKG we constructed contains rich knowledge, especially both TCM properties and clinical treatment of herbs that help to learn rich KE vectors of herbs and targets. TMKG is a foundational knowledge graph that can be further applied to various application scenarios, e.g. biomedical relationships inference (e.g. genes of diseases, symptoms, or syndromes), intelligent auxiliary diagnosis and treatment (e.g. disease diagnosis or drug/treatment recommendation). On the other hand, the residual-like graph convolution we designed can effectively capture the deep interactions among herbs and targets, capable of effectively integrating complex interaction data from different biomolecules; the BPR is a supervised module designed specifically for HTP. This supervised neural network framework does perform better on target prediction. Meanwhile, the flexible design of the HTINet2 framework is not only suitable for predicting drug targets but can also be extended to other graph-based bioinformatics problems such as disease gene identification and protein function prediction. We describe the process for applying HTINet2 to other tasks or datasets in the Section 2.5 of Supplemental Materials file 1. This wide applicability makes HTINet2 a versatile tool for various biomedical research applications.

There are still several work to do in the future. First, the current data of herb-target relationships are still noisy, how to maintain consistency and high quality with data from multiple sources remains a problem, and high-quality herb–target relationships remain a limitation. In the future, we will combine the two high-quality datasets SymMap [15] and SympGAN [48] built from our previous studies, integrating technologies such as entity alignment and semantic disambiguation to enhance data quality, to form a large-scale, high-quality data set of herb targets. Second, our HTINet2 is still a two-stage, not an end-to-end, framework, where the optimization objective (i.e. loss function) can optimize the neural network parameters of residual-like GCN, but cannot optimize the parameters of KE models. In the future, we will design an end-to-end unified framework, reducing redundancy and improving performance. Third, most neural network models are black boxes, making interpretability a critical and challenging issue, especially in the field of bioinformatics [49]. In the future, we aim to incorporate explainable models and techniques to facilitate a deeper understanding of the decision-making processes of the models [50]. Finally, the clinical translation process generally takes several years. We are committed to shortening this process or making it more efficient through computational biology, thereby narrowing the scope of potential drug targets. In the future, we will conduct wet experiments to validate the predicted results of HTINet2, to discover new and reliable herb targets.

**Key Points**

- We proposed a deep learning-based target prediction framework termed HTINet2, which designed three key modules, namely, TCM and clinical KGE, residual graph representation learning, and supervised target prediction.
- We constructed a large-scale knowledge graph that covers the TCM property and clinical treatment knowledge of herbs, and designed a component of deep KE to learn the deep KE of herbs and targets.
- We designed a residual-like graph convolution network to capture the deep interactions among herbs and targets, and a BPR loss to conduct supervised training and target prediction.
- Comparison experiment indicated the excellent performance of HTINet2 (HR@10 increased by 122.7% and NDCG@10 by 35.7%), ablation experiments illustrated the positive effect of our designed modules of HTINet2, and the case study demonstrated the reliability of the predicted targets of Artemisia annua and Coptis chinensis based on knowledge base, literature, and molecular docking.

## Abbreviations

Below are the abbreviations and full names used in this study.

1. TCM Traditional Chinese Medicine
2. HTI Herb-Target Interaction
3. HTP Herb-Target Prediction
4. PPI protein–protein interaction
5. TMKG Knowledge graph of TCM and western medicine
6. IES Inferred Evidence Score
7. GO Gene Ontology
8. GCN Graph Convolutional Network
9. BPR Bayesian Personalized Ranking
10. KE Knowledge Embedding
11. KGE Knowledge Graph Embedding
12. HR hit ratio
13. NDCG normalized discounted cumulative gain
14. SC silhouette score

## Supplementary Material

Supplemental_materials_file_1_bbae414

Supplemental_materials_file_2_bbae414

## Data Availability

The code of this study is available on the Github repository: https://github.com/2020MEAI/HTINet2.
