# Supplementary material for: HTINet2: herb–target prediction via knowledge graph embedding and residual-like graph neural network
Source: Brief Bioinform. 2024 Aug 23;25(5):bbae414. doi: 10.1093/bib/bbae414 (PMC11341278; doi:10.1093/bib/bbae414)
Supplement: Supplemental_materials_file_1_bbae414 [file supplemental_materials_file_1_bbae414.pdf]

Supplementary Materials for HTINet2: Herb Target Prediction via Knowledge Graph Embedding and Residual-like Graph Neural Network

1. Supplemental Materials and Methods

1.1 Knowledge graph of TCM and western medicine (TMKG)

The TMKG we constructed comes from multiple sources. Data derived from medical knowledge bases are more standardized, while data from traditional Chinese medicine classics are more complex. To ensure the quality of the knowledge graph we constructed, we performed data cleansing at the initial stage to enhance data quality. This included using scripts or programs, as well as inviting medical experts to review and correct the dataset. Below is a detailed description:

1. Selection of Data Sources: We carefully chose well-recognized and reliable data sources such as SymMap[1] and soFDA[2]. The quality of these data sources has been widely validated and utilized.

2. Data Preprocessing Using Programs or Scripts: Before constructing the knowledge graph, we standardized the datasets using data cleansing methods from natural language processing to maintain consistency across multiple sources. This included the following tasks:

a. Segmentation: For sentences in the raw dataset containing multiple entities, we used programs to segment the entities based on delimiters. (For example, in cases where a prescription contains multiple herbs or where one prescription corresponds to multiple symptoms, we split them into corresponding one-to-one relationships.)

b. Reorganization: For relationships described between entities in the raw dataset, we used scripts to batch add or remove entities based on the descriptions. (For example, adjusting the components of prescriptions.)

c. Replacement: To address variations in the representation of the same entity

across different sources, we used a "synonym table" to unify naming and perform deduplication.

d. Other Adjustments: We handled other regular issues encountered during standardization using programs after consulting with medical experts.

3. Review, Correction, and Quality Control by Medical Experts: After completing the preliminary work, we invited medical experts (from hospitals and universities) to review and correct the datasets processed by the programs. They also manually standardized issues that programs could not handle, such as consistency and ambiguity problems (e.g., normalizing "symptoms" in the dataset by removing descriptive terms and matching them to standard names). Finally, we conducted multiple rounds of quality checks on the knowledge graph to ensure the accuracy and completeness of the information.

The final statistics of entities and relationships of the TMKG are presented in Table1.

Table 1. Statistics of entities and relationships of TM KG

Head entities	Relationships	Tail entities	Number of head entities	Number of relationships	Number of tail entities
herb	herb_has_efficacy	efficacy	729	3207	377
herb	herb_treats_symptom	symptom	698	21476	2285
herb	herb_treats_syndrome	syndrome	596	2110	214
herb	herb_classified_as_type	type	699	699	48
herb	herb_classified_as_meridian	meridian	654	1550	12
herb	herb_classified_as_property	property	694	694	9
herb	herb_classified_as_taste	taste	694	1194	19
herb	herb_includes_ingredient	ingredient	686	74606	26060
herb	treatherb_treats_disease	disease	579	2163	323
prescription	prescription_includes_herb	herb	5271	56066	2716
prescription	prescription_has_efficacy	efficacy	1948	7474	593
symptom	contain	syndrome	6208	17398	864
symptom	symptom_alias_of	symptom	3568	16357	3568

ingredient	ingredient_actions_on_protein	protein	8490	142959	11540
disease	disease_has_associated_gene	gene	9711	256826	18737
protein	interacts_with	protein	17185	841068	17185
protein	gene_is_element_in_pathway	pathway	7251	25813	316
protein	gene_is_element_in_GO	GO	17549	61636	1207
I_syndrome	syndrome_has_subclass	II_syndrome	5	28	28
II_syndrome	syndrome_has_subclass	III_syndrome	28	240	240
prescription	herb_treats_symptom_cluster	symptom_cluster	4095	4217	3851
Symptom_cluster	symptom_cluster_includes_symptom	symptom	3851	56284	6296
prescription	prescription_treats_syndrome_set	syndrome_set	1512	1530	815
syndrome_set	syndrome_set_includes_syndrome	syndrome	815	1225	939
herb	herb_alias_of	herb	1814	2698	1813
prescription	prescription_alias_of	prescription	24	28	28

1.2 Knowledge embedding learning of TMKG

We utilized various embedding learning methods to represent entities in TMKG. To explain the main concept of network embedding algorithms, we took DeepWalk as an example. Given a graph G=(V, E), where V represents the set of vertices, and E⊆(V×V) represents the set of edges. The DeepWalk algorithm consists of two main components, as demonstrated below:

DeepWalk(G, w, d, γ, t)

Input: graph G(V, E)
window size w, embedding size d, walks per vertex γ, walk length t
Output: matrix of vertex representations $\Phi \in \mathbb{R}^{|V| \times d}$

Initialization: Sample Φ from $U^{|V| \times d}$
Build a binary Tree T from V
for i = 0 to γ **do**
    $O = Shuffle(V)$
    for each $v_i \in O$ ∈ **do**
        $W_{v_i} = RandomWalk(G, v_i, t)$
        SkipGram(Φ, W_{v_i}, w)
    end for
end for

SkipGram(Φ, W_{v_i}, w)

for each $v_j \in W_{v_i}$ **do**
    for each $u_k \in W_{v_i}[j - w : j + w]$ **do**
        $J(\Phi) = -log\Pr(u_k|\Phi(v_j))$
        $\Phi = \Phi - \alpha * \frac{\partial J}{\partial \Phi}$
    end for
end for

1.3 Experimental settings

CN is widely utilized as a baseline method for link prediction across various networks [1], positing that the greater the number of shared neighbors between two nodes, the higher the likelihood of an edge existing between them. The Salton [4] metric quantifies the proportion of common neighbors between two nodes relative to the total number of neighbors each node has. A high cosine similarity indicates a certain degree of overlap among the neighbors of the nodes, suggesting similarity between them. The Jaccard index [5] is typically defined as the size of the intersection of two sets relative to the size of their union's complement. In link prediction, the Jaccard distance measures the number of shared neighbors between two nodes to assess the potential existence of a link between them. HPI refers to nodes in a network that connect to many other nodes [6], often denoting higher influence and significance. LHN-1 measures the likelihood of a link between two vertices based on the ratio of the number of common neighbors to the product of their degrees, where a value closer to 1 indicates a higher probability of link existence [7]. The AA index assigns a weight to each shared neighbor based on the degree of common neighbor, then summing up these contributions [8]. Inspired by the AA index, Zhou et al. [9] designed the RA index, as shown in formula 1, which is predicated on simulating the process of resource allocation.

$$s_{xy} = \sum_{z \in \Gamma_{(x)} \cap \Gamma_{(y)}} \frac{1}{k_z} \quad (1)$$

The heNetRW constructs a heterogeneous herb-target network [10], then simulates a random walk algorithm on the network to identify protein targets of herbs. Prince is a

network-based method for prioritizing disease genes and inferring protein complex associations [11]. It is based on formulating constraints on the prioritization function that relate to its smoothness over the network and usage of prior information.

During the Knowledge Embedding Learning of TMKG phase, we employed multiple network embedding methods, including DeepWalk, LINE, node2vec, GraRep, and HOPE. The hyperparameter settings for these methods are as follows: DeepWalk (window size: 10, number of walks: 10, walk length: 80), LINE (negative ratio: 5, order: 3), and node2vec (window size: 10, number of walks: 10, walk length: 80, with both q and p set at 1.0).

2. Supplemental Results

2.1 Silhouette score for TMKG entities

Different types of entities, such as herb, protein, symptom and disease, etc. are grouped into different clusters. In assessing the coherence of these clusters, we calculated their Silhouette score (SC). The SC ranges from -1 to 1, with values closer to 1 indicating more coherent clustering. The results show there is highest clustering in cluster results of GraREP (SC=0.0334), low clustering in that of other embedding methods (SC=-0.0302 for LINE, SC=-0.1377 for node2vec and SC=-0.2336 for HOPE).

2.2 Performance comparison of different Knowledge Embedding methods

In the experiment, we conducted comparative experiments with various Embedding Learning algorithms. The specific performance outcomes are shown in Table 2.

Table 2. Performance comparison of different Knowledge Embedding Learning methods

KG embedding models	Top@1		Top@3		Top@5		Top@10	
	HR	NDCG	HR	NDCG	HR	NDCG	HR	NDCG
LINE	0.3556	0.3556	0.3403	0.3356	0.3669	0.3447	0.4332	0.3704
GraRep	0.3598	0.3598	0.3389	0.3392	0.3673	0.3468	0.4382	0.3745
node2vec	0.3494	0.3494	0.3403	0.3353	0.3687	0.3458	0.4431	0.3736
DeepWalk	0.3536	0.3536	0.3448	0.3398	**0.3706**	**0.35**	**0.4458**	0.3746
HOPE	**0.3598**	**0.3598**	**0.3469**	**0.3439**	0.3688	0.3477	0.4354	**0.3753**

2.3 Hyper-parameter sensibility of HTINet2

In the parameter sensitivity experiments, we initially conducted comparative experiments on the number of GCN layers in the Graph Representation Learning component. The specific performance outcomes are shown in Table 3. Subsequently, we conducted experiments on adjusting the dimensions of the embedding vectors, with the specific performance detailed in Table 4.

Table 3. HTINet2's performance with different numbers of GCN layers.

Layers	Top@1		Top@3		Top@5		Top@10	
	HR	NDCG	HR	NDCG	HR	NDCG	HR	NDCG
0	0.3347	0.3347	0.3128	0.3137	0.3313	0.3135	0.3968	0.3381
1	0.3515	0.3515	0.3222	0.3222	0.3423	0.3308	0.4125	0.3572
2	0.3368	0.3368	0.3145	0.3145	0.3372	0.3195	0.4112	0.348
3	0.3452	0.3452	0.311	0.3156	0.336	0.3229	0.4063	0.3484
4	0.3494	0.3494	0.3166	0.3196	0.3388	0.3243	0.4091	0.3514
5	0.3347	0.3347	0.3229	0.3219	0.3358	0.3217	0.4109	0.3511
6	0.341	0.341	0.3253	0.3251	0.3323	0.3224	0.4034	0.3493

Table 4. HTINet2's performance with different dimensions of the embedding of nodes.

Dimensions	Top@1		Top@3		Top@5		Top@10	
	HR	NDCG	HR	NDCG	HR	NDCG	HR	NDCG
32	0.3159	0.3159	0.3023	0.3016	0.3243	0.3048	0.3931	0.3294
64	0.3452	0.3452	0.311	0.3156	0.336	0.3229	0.4063	0.3484
96	0.3515	0.3515	0.3264	0.3273	0.3493	0.3334	0.4167	0.3582
128	0.3556	0.3556	0.3278	0.3302	0.3566	0.3364	0.4249	0.3636
256	0.3536	0.3536	0.3333	0.3323	0.3645	0.3429	0.4335	0.3689
512	0.3556	0.3556	0.3389	0.3389	0.3663	0.3447	0.435	0.3727

Finally, in the Knowledge Embedding Learning of TMKG stage, we adjusted the window size of the DeepWalk algorithm and observed its impact on the performance of HTINet2, the results are presented in Table 5. The results indicate that the model does exhibit sensitivity to window size changes, yet it remains robust within a generally

acceptable range. As the window size increases, the overall performance of the model exhibits a declining trend. However, there is a slight rebound in performance when the window size is set to 10, achieving a performance of HR@10 at 0.4458 and NDCG@10 at 0.3746. We have identified an optimal window size 10 that balances performance and computational efficiency.

Table 5. Performance of HTINet2 by different window size

Window size	Top@1		Top@3		Top@5		Top@10	
	HR	NDCG	HR	NDCG	HR	NDCG	HR	NDCG
1	**0.3682**	**0.3682**	0.3375	0.3382	0.3503	0.3374	0.4217	0.3642
3	0.3536	0.3536	0.3309	0.3287	0.342	0.3323	0.4121	0.3576
5	0.3431	0.3431	0.326	0.3261	0.3419	0.3286	0.4103	0.3543
7	0.3389	0.3389	0.3257	0.3255	0.3404	0.3301	0.4057	0.3533
10	0.3536	0.3536	**0.3448**	**0.3398**	**0.3706**	**0.35**	**0.4458**	**0.3746**

2.4 Case study

As shown in Figure 1 A1-A3, the results of docking analysis of Artemisia annua and its targets not listed in the main text, namely MC1R, NR3C1, and CD86, are displayed. Similarly, as illustrated in Figure 2 B1-B3, the results of docking analysis of Coptis chinensis and its targets not mentioned in the main text, namely POR, PPARA, and PRSS3, are presented.

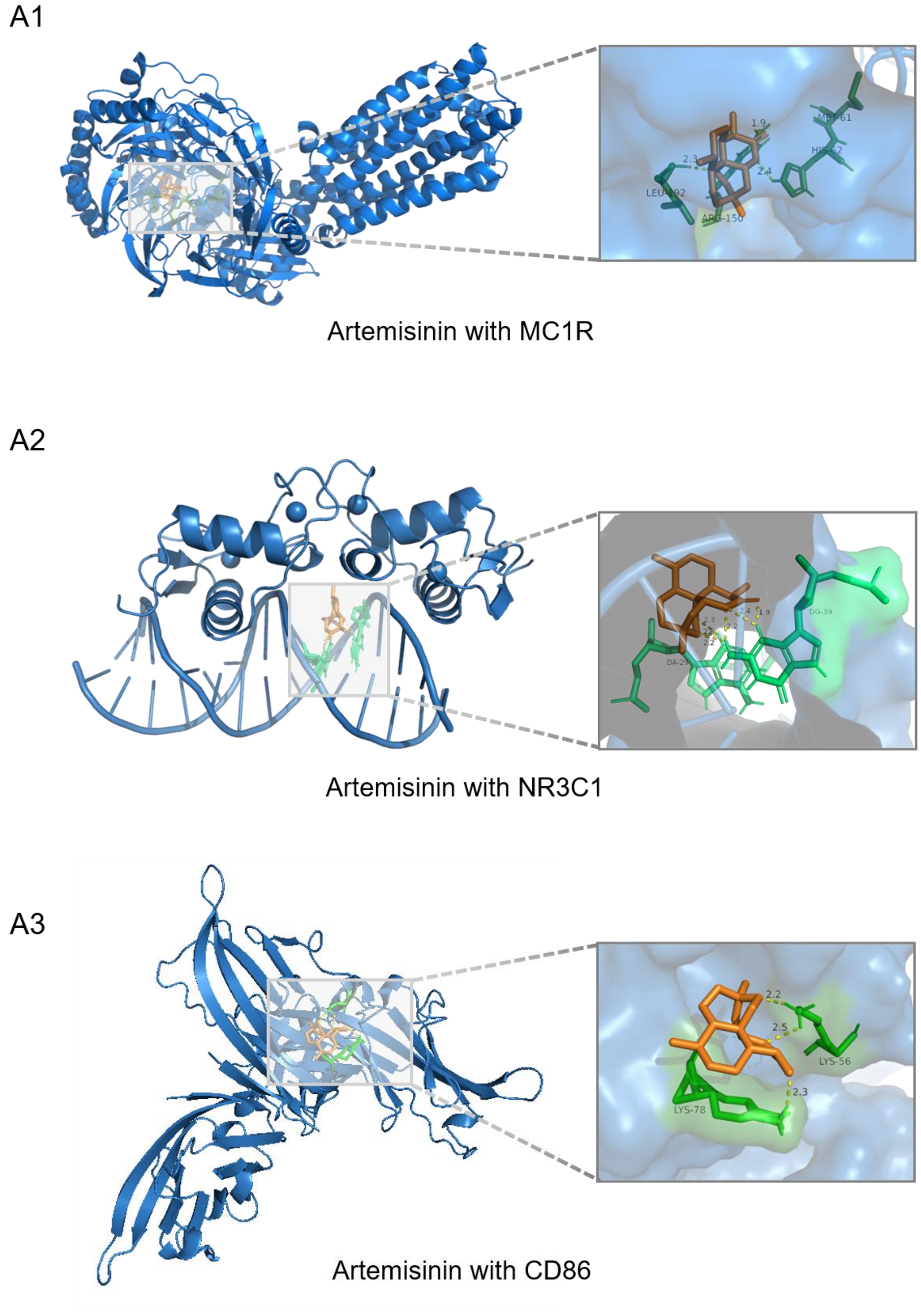

Figure 1. 3D diagrams of molecular docking results. **(A1-A3)** Artemisinin with MC1R, NR3C1 and CD86.

B1

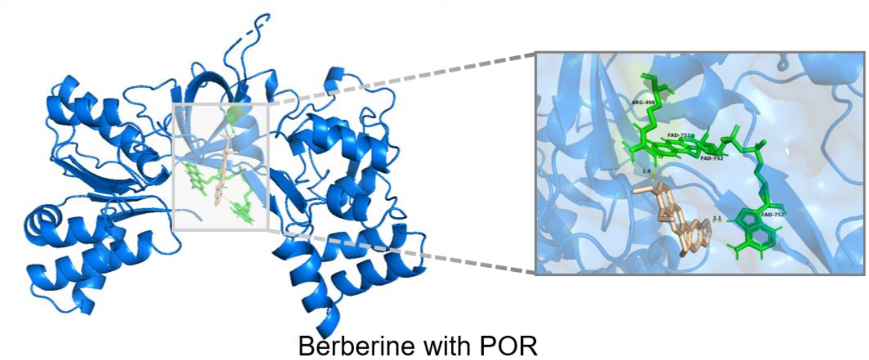

Berberine with POR

B2

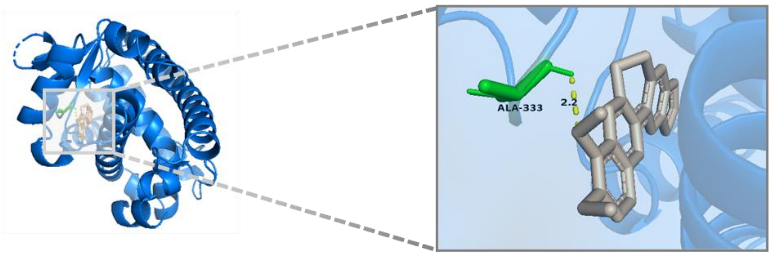

Berberine with PPARA

B3

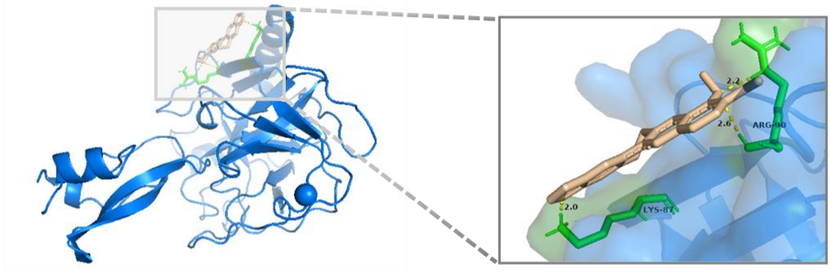

Berberine with PRSS3

Figure 2. 3D diagrams of molecular docking results. **(B1-B3)** Berberine with POR, PPARA and PRSS3.

2.5 Performance of HTINet2 on other datasets

Our model is a general-purpose framework that can be used for link prediction tasks on other datasets. The specific steps are as follows:

1.Data Preprocessing: Initially, the dataset requires necessary preprocessing, which includes data cleaning, formatting, and normalization. For instance, when using the IMPPAT database, it is essential to extract and transform data related to plants and phytochemicals. The format should be converted such that the first column represents plant identifiers, while subsequent columns represent phytochemical identifiers. Depending on experimental requirements, the dataset is divided into training, testing, and validation sets proportionally to meet the input requirements of the HTINet2 model.

2.Knowledge Embedding Learning: If the new task domain is similar to the domain of our study, the TMKG knowledge graph can be directly utilized for Knowledge Embedding Learning to obtain initial vectors. If not, it may be necessary to reconstruct a knowledge graph suitable for the new dataset to proceed with Knowledge Embedding Learning. However, this step is optional.

3.Graph Structure Construction: Based on the features extracted through Knowledge Embedding Learning and the biological relationships to be predicted, a graph structure is constructed. In this structure, nodes may represent drugs, targets, or disease genes, while edges represent their interactions or associations.

4.Model Training and Tuning: The HTINet2 model is trained using the prepared graph structure data. During this process, model parameters such as number of layers can be adjusted to optimize performance and prevent overfitting.

5.Validation and Testing: Once training is complete, the model undergoes validation and testing to assess its performance on the new dataset. This stage is crucial for determining the efficacy and reliability of the model in new applications.

***Performance of HTINet2 on IMPPAT dataset*:** We selected associations between plant-part-phytochemical from the IMPPAT database. These data were divided into training, testing, and validation sets at a ratio of 8:1:1, respectively. It includes 4,010 Indian medicinal plants and 17,967 phytochemicals. Experiments were conducted on

our model, HTINet2, and the results are presented in the Table 6. The results indicate that on the IMPPAT dataset, the model achieved a performance of 0.4174 in HR@10 and 0.3421 in NDCG@10.

Table 6. Performance of HTINet2 on IMPPAT dataset

	Top@1		Top@3		Top@5		Top@10	
	HR	NDCG	HR	NDCG	HR	NDCG	HR	NDCG
HTINet2	0.293	0.293	0.3369	0.3152	0.3638	0.3248	0.4174	0.3421

References

[1] Wu Y, Zhang F, Yang K, et al. SymMap: an integrative database of traditional Chinese medicine enhanced by symptom mapping[J]. Nucleic Acids Research, 2019, 47(D1): D1110-D1117.

[2] Zhang Y, Wang N, Du X, et al. SoFDA: an integrated web platform from syndrome ontology to network-based evaluation of disease-syndrome-formula associations for precision medicine[J]. Science Bulletin, 2022, 67(11): 1097-1101.

[3] Lü L, Zhou T. Link prediction in complex networks: A survey[J]. Physica A: Statistical Mechanics and its Applications, 2011, 390(6): 1150-1170.

[4] Salton G. Introduction to modern information retrieval[J]. McGraw-Hill, 1983.

[5] Jaccard P. Étude comparative de la distribution florale dans une portion des Alpes et des Jura[J]. Bull Soc Vaudoise Sci Nat, 1901, 37: 547-579.

[6] Ravasz E, Somera A L, Mongru D A, et al. Hierarchical organization of modularity in metabolic networks[J]. Science, 2002, 297(5586): 1551-1555.

[7] Leicht E A, Holme P, Newman M E J. Vertex similarity in networks[J]. Physical Review E, 2006, 73(2): 026120.

[8] Adamic L A, Adar E. Friends and neighbors on the web[J]. Social Networks, 2003, 25(3): 211-230.

[9] Zhou T, Lü L, Zhang Y C. Predicting missing links via local information[J]. The European Physical Journal B, 2009, 71: 623-630.

[10] Yang K, Liu G, Wang N, et al. Heterogeneous network propagation for herb target identification[J]. BMC Medical Informatics and Decision Making, 2018, 18: 27-37.

[11] Vanunu O, Magger O, Ruppin E, et al. Associating genes and protein complexes with disease via network propagation[J]. PLoS Computational Biology, 2010, 6(1): e1000641.
